# Bacterial Fertilizers Based on *Rhizobium laguerreae* and *Bacillus halotolerans* Enhance *Cichorium endivia* L. Phenolic Compound and Mineral Contents and Plant Development

**DOI:** 10.3390/foods10020424

**Published:** 2021-02-15

**Authors:** Alejandro Jiménez-Gómez, Ignacio García-Estévez, M. Teresa Escribano-Bailón, Paula García-Fraile, Raúl Rivas

**Affiliations:** 1 Departamento de Microbiología y Genética, Universidad de Salamanca, Edificio Departamental de Biología, 37007 Salamanca, Spain; paulagf81@usal.es (P.G.-F.); raulrg@usal.es (R.R.); 2 Spanish-Portuguese Institute for Agricultural Research (CIALE), 37185 Salamanca, Spain; 3 Grupo de Investigación en Polifenoles (GIP), Departamento de Química Analítica, Nutrición y Bromatología, Faculty of Pharmacy, Universidad de Salamanca, 37007 Salamanca, Spain; igarest@usal.es (I.G.-E.); escriban@usal.es (M.T.E.-B.); 4 Associated Unit USAL-CSIC (IRNASA), 37008 Salamanca, Spain

**Keywords:** phenolic acids, escarole, bioactive compounds, biofertilizer, flavonols

## Abstract

Today there is an urgent need to find new ways to satisfy the current and growing food demand and to maintain crop protection and food safety. One of the most promising changes is the replacement of chemical fertilizers with biofertilizers, which include plant root-associated beneficial bacteria. This work describes and shows the use of *B. halotolerans* SCCPVE07 and *R. laguerreae* PEPV40 strains as efficient biofertilizers for escarole crops, horticultural species that are widely cultivated. An in silico genome study was performed where coding genes related to plant growth promoting (PGP) mechanisms or different enzymes implicated in the metabolism of phenolic compounds were identified. An efficient bacterial root colonization process was also analyzed through fluorescence microscopy. SCCPVE07 and PEPV40 promote plant development under normal conditions and saline stress. Moreover, inoculated escarole plants showed not only an increase in potassium, iron and magnesium content but also a significant improvement in protocatechuic acid, caffeic acid or kaempferol 3-*O*-glucuronide plant content. Our results show for the first time the beneficial effects in plant development and the food quality of escarole crops and highlight a potential and hopeful change in the current agricultural system even under saline stress, one of the major non-biological stresses.

## 1. Introduction

The current agricultural system is based on the application of large quantities of chemical fertilizers that supply around 50% of the necessary nutrients for plant development [1]. However, the extensive use of this type of fertilization can lead to a wide range of environmental problems such as soil deterioration, the imbalance of nutrient proportions or water pollution. Therefore, the application of more eco-friendly fertilizers replacing the chemical ones has been considered one of the main agricultural challenges in recent years.

For several decades, many scientific projects have focused on plant growth promoting rhizobacteria (PGPR), one group of microorganisms that cannot only colonize the rhizosphere of crops but also improve their development through direct and indirect mechanisms [2]. Apart from enhancing plant development, PGPR bacteria are also nowadays being described as increasing food quality and improving the plant mineral content and bioactive compound concentrations [3], highly reported for its beneficial effects in human health [4].

As diets rich in fruit and vegetables are associated with a decrease in cardiovascular diseases and cancer, vegetables such as escarole are consumed in increasing amounts due to the beneficial effects for human health [5]. Escarole (*Cichorium endivia* L.) is attributed to contain antioxidant compounds, vitamins and phenolic compounds, which have been described as having a significant antioxidant activity [6] and therefore being one of the most economically important vegetables. Phenolic compounds are secondary metabolites that are synthesized in plants through the shikimic acid and phenylpropanoid pathways. They can be divided into two main families, flavonoids (which show a C6–C3–C6 general structural backbone) and non-flavonoids, among which phenolic acids are one of the most important groups in vegetables [7]. The direct scavenging of free radicals and reactive oxygen species (ROS) that can be attributed to these compounds is the main mechanism described for explaining their health protecting effects [8]. Moreover, these compounds are also important for food quality because they are closely related to food sensory characters such as bitterness, astringency and herbaceous flavors, thus affecting consumer acceptance [9].

Even though every year there are more published studies that increase the knowledge of PGPR bacteria effects in food quality, these improvements are still scarcely reported under abiotic stress conditions such as soil salinity, one of the major non-biological stresses [10]. Soil salinity severely limits plant development and affects agricultural productivity causing significant biochemical and physiological changes in crops. It affects plant growth and seed germination rates and induces water and oxidative stresses and nutritional disorders [11]. 

Scientific estimations calculate that soil salinity affects more than 800 million hectares of land worldwide and it is significantly increasing every year. Problems resulting from the augmentation in soil salinity are expanding worldwide because of global climate change, which has a great impact on the agricultural economy that has been growing for decades [10].

On the other hand, bacteria applied to fields as biofertilizers must be innocuous for human and environmental health and satisfy all food safety requirements [12], particularly on vegetables that are consumed raw such as escarole.

It must be remarked that bacterial strain effects vary from laboratories to the field and between different plant species due to an important host specificity. Some PGPR strains show a high potential to promote the growth of a particular crop and have no response in others [13]. *Bacillus halotolerans* and *Rhizobium laguerreae* are two bacterial species that have received the most extensive attention due to in vitro mechanisms and physiological traits and they have been previously reported for their beneficial plant effects in other crops of agri-food interest [11,14]. Related to the *Bacillus* genus, there are a few transcriptomic analyses that describe the genes involved in plant growth promotion and in biofilm formation [15] as well as in the production of secondary metabolites such as phenolic compounds or osmolytes [11]. Within the variety of species of the *Rhizobium* genus, the species *R*. *laguerreae* has been widely studied. We found several studies analyzing its interaction with legume plants. These strains formed biofilms and produced acyl-homoserine lactones (AHLs) involved in the quorum sensing regulation process [16]. They also solubilize phosphate, produce siderophores and symbiotic atmospheric nitrogen fixation [16]. Moreover, Ayuso-Calles and collaborators [17] reported a production of enzymes related to plant food quality such as derivatives of caffeoyl acid and quercetin. The aim of this study was to analyze for the first time the effects on phenolic composition, mineral content and plant growth development of escarole plants after the inoculation of probiotic bacterial strains from *Bacillus halotolerans* and *Rhizobium laguerreae* species under saline conditions.

## 2. Materials and Methods

### 2.1. Bacterial Strains

The bacterial strains used in this work were previously isolated from nodules of *Phaseolus vulgaris*. They were classified within the species *Rhizobium laguerreae* PEPV40 [14] and *Bacillus halotolerans* SCCPVE07 [11] by 16S rRNA gene analysis.

In this study, the PEPV40-GFP (green fluorescence protein), which contains the plasmid phC60, was used in fluorescence microscopy experiments. The PEPV40-GFP strain was also obtained in Jiménez-Gómez et al. [14].

PEPV40, PEPV40-GFP and SCCPVE07 were routinely grown at 28 °C in YMA (yeast manitol agar), TY (tryptone yeast, supplemented with 10 µg/mL tetracycline) and TSA (tryptic soy agar), respectively, for five days for *Rhizobium* strains and one day for *Bacillus*.

### 2.2. Draft Bacterial Genome Sequencing and Annotation

Bacterial genomic DNA was obtained from pure colonies of the PEPV40 strain grown on YMA plates and collected after 24 h at 28 °C using the ZR Fungal/Bacterial DNA MiniPrep (Zymo Research). The draft genome sequence of the bacterial isolates was obtained by shotgun sequencing on an Illumina MiSeq platform via a paired-end run (2 × 251 bp). The sequence data was assembled by Velvet 1.2.10 and gene annotation was performed using RAST 2.0 (Rapid Annotation using Subsystem Technology) [18].

The draft genome sequence of the strain *Rhizobium laguerreae* PEPV40 was deposited in GenBank under the accession number JABWPR000000000. The draft genome sequence of the strain *Bacillus halotolerans* SCCPVE07 was obtained previously in Jiménez-Gómez et al. [11].

### 2.3. Bacterial Colonization of Escarole Roots

Escarole seeds (*Cichorium endivia* L.) were surfaced-sterilized with a solution of HgCl_2_ 0.1% for four minutes. This type of sterilization was also used for in vitro and greenhouse experiments. The seeds were then washed five times with sterile water and germinated on water-agar plates. Seedlings were kept in the dark at 28 °C for two days and then transferred to 1.5% agar square plates (12 × 12 cm) with a distribution of six seedlings per plate.

According to the bacterial strain applied, the colonization assays consisted of two different treatments. On the one hand, escarole roots were inoculated with PEPV40-GFP and observed directly at seven and fourteen days post inoculation (dpi). On the other hand, escarole roots inoculated with the SCCPVE07 strain were immersed in an Uricase antibody preparation following the instructions described in Jiménez-Gómez et al. [11] before microscopy analysis, which was also carried out at seven and fourteen dpi.

To prepare the PEPV40-GFP bacterial suspension, the strain was grown for five days at 28 °C on TYTc^10^ plates. The SCCPVE07 strain was grown for one day at 28 °C on TSA plates. Both suspensions were adjusted to an OD (optical density) (600 nm) of 0.6, which corresponded with a final concentration of 10^8^ CFU/mL after counting the number of viable cells using the serial decimal dilution method.

Propidium iodide of 10 μm was added into the roots of both treatments 10 min before microscopy root observations. Fluorescence microscopy was carried out with a Nikon Eclipse 80i fluorescence microscope and a mercury lamp was used for green fluorescent protein excitation.

### 2.4. Effects of Bacterial Strains on Plant Seedlings

Surfaced-sterilized escarole seedlings were transferred to 0.2 dm^3^ pots that contained sterilized vermiculite “SEED PRO 6040” (PROJAR, Madrid, Spain) as a substrate. The escarole plants were inoculated with 2 mL of bacterial suspension at 10^8^ CFU mL^−1^ (0.6 OD measured at 600 nm). Fifty-four pots were used as a negative control and fifty-four pots were added per each bacterial suspension. In each treatment half of the plants were irrigated with water from a bottom reservoir every 48 h and the other half with a saline condition (NaCl 100 mM). The pots were maintained in a growth chamber. Fifteen days post inoculation, the data of fresh weight per plant, dry weight per plant and leaf size were recorded. The experiment was performed at least three times. Therefore, 81 plants per treatment and growth condition were collected for obtaining the data.

### 2.5. Evaluation of Escarole Growth Promotion and Nutritional Content

The ability to promote plant growth was investigated on escarole plants using a mix of 2.4 L of non-sterilized soil and vermiculite (3:1 v/v) as a substrate in plastic pots.

The escarole seeds were surface-sterilized and pre-germinated. They were transferred into the substrate and inoculated with 5 mL of bacterial suspension with a final concentration of 10^8^ CFU mL^−1^ (0.6 OD measured at 600 nm). Uninoculated escarole plants were included as negative controls under the same conditions. The plants were watered from a bottom reservoir every 48–72 h depending on plant demand. The solution of NaCl 100 mM (to obtain saline conditions) was added in the specific treatments. The plants were maintained in an illuminated greenhouse (night temperatures ranging from 15 to 20 °C and day temperatures ranging from 25 to 30 °C) with a humidity control for 60 days. A total of 18 plants were used in each treatment. The values of the number of leaves, shoot fresh weight and shoot dry weight per plant were recorded. 

The dry plants were used for the analysis of N, C, P, Mg, K and Fe contents. The analysis was performed by the Ionomics Service at CEBAS-CSIC (Murcia, Spain), using elemental analyst model TruSpec CN628 equipment for the N analysis and ICP THERMO ICAP 6500DUO equipment for the analysis of the remaining elements.

### 2.6. Evaluation and Analysis of Phenolic Compound Contents

After 60 days post bacterial inoculation, the phenolic composition of the plant material was determined as previously reported [11]. Freeze-dried samples (5 mg) were extracted in a bath of ultrasound using MeOH:H_2_O (80:20). An extraction was performed three times and supernatants were combined and cleaned by liquid-liquid extraction with hexane. Organic solvents were removed under reduced pressure until a final volume of 2 mL was reached.

HPLC-DAD-MS analyses were performed to determine the phenolic composition of the extracts by using a Hewlett–Packard 1200 series liquid chromatograph (Agilent Technologies, Waldbronn, Germany). Chromatography was carried out in a Spherisorb® S3 ODS-2 C18 reversed phase 3 μm 150 × 4.6 mm column (Waters Corporation, Milford, MA, USA) thermostatted at 35 °C using formic acid (0.1 mL L^−1^) and acetonitrile (B) as solvents [11]. The preferred wavelengths employed for detection were 280, 330 and 370 nm and full UV-vis spectra were recorded from 220 to 600 nm. Mass spectrometry was carried out using an API 3200 Qtrap (Applied Biosystems, Darmstadt, Germany) equipped with an Electrospray Ionization (ESI) source and a triple quadrupole-ion trap mass analyzer and employed a previously reported methodology [11]. Spectra were recorded in negative ion mode between *m*/*z* 100 and 1700 and both full scan and MS/MS analyses were performed.

The chromatographic retention times, UV-vis spectra and mass spectra (*m*/*z* and fragmentation patterns) were used for compound identification. The quantification was performed by an external standard method from the peak area values obtained in the chromatograms recorded at 280 nm (protocatechuic acid derivative), 330 nm (caffeic acid and its derivatives including cichoric acid) or 360 nm (flavone and flavonol derivatives). The content of protocatechuic acid and caffeic acid derivatives were expressed as gallic acid and caffeic acid equivalents, respectively. Kaempferol and quercetin derivatives were expressed as kaempferol 3-*O*-glucuoside and quercetin 3-*O*-glucoside equivalents, respectively. The extraction and analysis were performed in triplicate and the results provided are the mean values expressed in g Kg^−1^ of plant dry weight. 

### 2.7. Statistical Analysis

The statistical analysis was performed using the software program StatView 5.0 (SAS Institute Inc., Cary, NC, USA). An analysis of variance (ANOVA) followed by Fisher’s protected least significant difference test (Fisher’s PLSD) were performed as the statistical analysis.

## 3. Results and Discussion

### 3.1. Bacterial Genome Mining

Nowadays, bacterial genome mining strategies and studies are considered a useful tool to identify functionally bacterial properties that provide further insights into plant-microbe interactions and new biofertilizer formulations [19].

Although many mechanisms have been described to explain plant growth promotion by bacteria, a single mechanism is not responsible for the full positive effects. Several mechanisms rather than just one participate in the beneficial association [20]. 

As described in Jiménez-Gómez et al. [11], the SCCPVE07 strain solubilizes phosphate and produces siderophores, exopolysaccharides and phytohormones. Moreover, a wide variety of enzymes involved in flavonoid and phenolic acid metabolisms were reported. In this study, it was observed that apart from the previously published work the SCCPV07 bacterial genome encoded the gene involved in aromatic amino acid aminotransferase (AAAAT) (EC 2.6.1.57) biosynthesis, which is a precursor of phenylalanine. Moreover, the gene that encodes the catechol-2,3-dioxygenase enzyme (EC 1.13.11.2) presented in the protocatechuic acid pathway was also reported. Both are interesting in the active compounds in plants [21].

Among the range of essential plant nutrients, phosphorus is a good example required in plant growth and development. Currently, phosphorus deficiency is one of the major issues and limitations to crop production. Furthermore, it is estimated that the phosphorus consumption in the agricultural system is increasing around 2.5% per year [22]. In this sense, the use of PGPR bacteria in the increase of phosphorus efficiency through making insoluble phosphorus compounds available for plants is currently a focus of special attention [11]. The *R. laguerreae* PEPV40 encodes several enzymes involved in the transformation of inorganic substrates into more easily assimilable chemical forms for plants such as pyrophosphatase (EC 3.6.1.1) or citrate synthase (EC 2.3.3.1). 

Microorganisms have evolved to produce siderophores, which are secreted to solubilize iron from their surrounding areas. Apart from to satisfy nutritional requirements, it is also a key point to cope with other bacteria in the plant rhizosphere. Phytopathogenic bacteria or fungi can be deprived of iron availability and therefore the production of antibiotics is extremely decreased [23]. The PEPV40 genome was observed to encode genes for iron acquisition and siderophore biosynthesis such as aerobactin. The biosynthesis above described led to a notable increase in iron and phosphorus plant content in the escarole leaves analyzed (Table 1).

According to the importance of the colonization process for a successful plant-microbe interaction, the mechanisms involved in bacterial root attachment were analyzed. The PEPV40 bacterial genome encodes genes involved in cellulose production [beta-1,4-glucanase (cellulase) (EC 3.2.1.4)], broadly described as essential in the colonization process.

The functional analysis of the production of phytohormones has been thoroughly investigated due not only to the agronomic impact on horticultural crops but also to the significant improvement in nutrient absorption. However, plants are not the only organisms with the ability to produce them.

Indol-3-acetic acid (IAA) is reported as one of the most influential phytohormones in enhancing plant growth promotion [20]. In this sense, the PEPV40 strain genome encodes genes related to the biosynthesis of indol-3-acetic acid (Indole-3-glycerol phosphate synthase (EC 4.1.1.48) and it also encodes AmiE acetamidases (EC 3.5.1.4) involved in the synthesis of IAA by the so-called indole-3-acetamide pathway by converting indole-3-acetamide to indole acetic acid.

As mentioned before, soil salinity is a worrying issue in agriculture today. Previous research studies have discovered that the plant accumulations of solutes such as choline or proline act as a natural defense system from salinity. In this sense, the application of microorganisms with the ability of this solutes production can be an effective and hopeful solution. The PEPV40 genome encodes *betA* and *betB* genes, which are involved in the codification of choline dehydrogenase (EC 1.1.99.1) and betaine aldehyde dehydrogenase (BADH), respectively. As it can be observed in Table 1, under salinity stress conditions the inoculation with the strain PEPV40 did not increase the sodium content and the results obtained in the control treatment and the plants inoculated with SCCPVE07 strain were similar.

As phenolic compounds have been related to both health protective effects and food quality [8,9], bacterial genes potentially involved in the biosynthesis or transport of phenolic compounds were also searched for in the PEPV40 genome. The PEPV40 genome shows a wide variety of important genes in flavonoid and phenolic acid metabolisms.

We searched for genes involved in the caffeic acid pathway, which is involved in lignin synthesis and interacts with reactive oxygen species. The biosynthesis of ferulic acid is carried out through caffeic acid whose process is mediated by the enzyme 3-*O*-methyltransferase [EC 2.1.1.68], which was not detected in the in silico analysis. However, several genes involved in previous steps in the synthesis route of phenylpropanoid compounds were detected such as the gene that encodes the enzyme cinnamoyl esterase. Furthermore, the gene encoding the enzyme trans-hydratase-feruloyl-CoA, involved in the production of vanillic acid from ferulic acid as a substrate, was also detected.

The presence of the enzyme’s aromatic amino acid aminotransferase (AAAAT) (EC 2.6.1.57) and EC 2.3.1.74 and a naringenin-chalcone synthase were also observed. The third one is involved in obtaining naringenin from phenylalanine, a flavone that acts as a precursor in the biosynthesis pathways of kaempferol, quercetin and apigenin [24].

Multiple bacterial strains of *Rhizobium* and *Bacillus* genera have also been previously described as containing a wide variety of genes involved in promoting plant growth in their genomes. Ambreetha and collaborators [25] reported a significant modification in the architecture of rice roots modulated by the expression of *Bacillus* auxin-responsive genes. On the other hand, Pérez-Montaño et al. [26] reported many *Rhizobium* genes that could represent a strategy to establish symbiosis under salt stressing conditions.

Based on the study described in Jiménez-Gómez et al. [11] and the results here presented, strains SCCPVE07 and PEPV40 exhibited an interesting potential as plant growth promoting microorganisms. However, these abilities were also checked and presented in planta even under salinity stress conditions with the following results.

### 3.2. Bacterial Colonization Analysis of Escarole Roots

**Figure 1:**
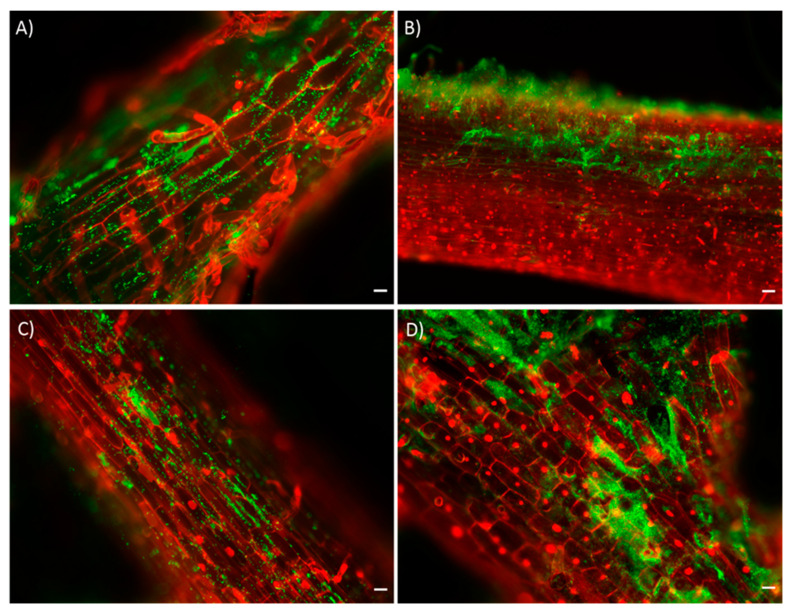
Bacterial colonization of escarole roots. Fluorescence optical micrographs of escarole seedlings roots inoculated with the PEPV40 strain (**A**,**C**) and SCCPVE07 (**B**,**D**) obtained at 7 (**A**,**B**) and 14 (**C**,**D**) days after inoculation. The micrographs show the bacterial ability (green color) to colonize the root surfaces, the base of emerging roots and the initiation of microcolonies. White bar of **A**, **C** and **D** images, 100 µM. Bar of **B** image, 200 µM.

It was observed that the PEPV40 strain colonized escarole root surfaces, forming microcolonies of biofilm initiation in the intercellular spaces in the cortical cells on both days of analysis (Figure 1 A–C). On the other hand, Figure 1 reveals that the SCCPVE07 strain (Figure 1 B–D) also colonized root surfaces and this was significantly so in the base of emergent lateral roots. Both strains increased the colonization gradually during the observations.

Bacterial root colonization is a key point required for PGPR bacteria. The adhesion is related to the production of a wide range of metabolites involved in plant-microbe interaction or to the ability to mobilize plant nutrients. Moreover, it is important in studies of bacterial persistence and understanding when microbes are applied in the soil [27]. In this sense, biofilm formation is recognized for its beneficial role in the PGP traits and the improvement in bacterial persistence in plant roots [28].

The colonization dynamics here reported have been previously described in other PGPR strains of the same genera. Our results showed that PEPV40 and SCCPVE07 not only presented the same distribution as other bacterial PGPR strains from *Rhizobium* and *Bacillus* genera, respectively [29,30], but also their colonization patterns were similar to other strains in different horticultural crop roots [11,27]. However, this study is the first to report *Rhizobium laguerreae* and *Bacillus halotolerans* strains as efficient colonizers of escarole roots.

### 3.3. Effects of Bacterial Strains on Escarole Growth and Nutritional Content

The results of escarole growth promotion in the in vitro and greenhouse experiments (Table 2) showed that PEPV40 and SCCPVE07 strains improved the growth parameters evaluated in non-stress and under salt stress conditions.

On the one hand, in the in vitro experiments the highest values were always shown in escarole plants inoculated with a bacterial treatment. In non-stress conditions, the stem length was increased by 19.9% and the shoot dry weight up to 14.3% in the case of the PEPV40 strain application compared with the respective control. However, under salt stress the best values were reported after the application of the SCCPVE07 strain. In particular, the largest increase (25.0%) was shown in the shoot fresh weight.

On the other hand, the results from the greenhouse experiments also showed that in a general way the best values were described in escarole plants after bacterial strain applications. In non-stress conditions, the highest growth values were always reported in plants inoculated with the PEPV40 strain. The number of leaves was significantly increased by 14.0% and the shoot fresh and dry weight were 18.1% and 17.6%, respectively, compared with the control. In the same way, under salt stress conditions and the SCCPVE07 strain, the escarole plants showed the best shoot fresh (76.0%) and dry (76.1%) values regarding the respective control treatment.

The results of the present study showed important improvements in nutrient plant content compared with their respective plants in the un-inoculated treatments.

The enhancement in the specific nutrient content was probably related to those genes encoded in bacterial genomes, previously described for their relation in the ability to mobilize plant nutrients. Most values were statistically higher after the bacterial application. However, the phosphorus, magnesium and iron showed the greatest improvements. The magnesium content showed up to 34.3% in non-saline conditions and 43.5% under saline stress both after PEPV40 application and compared with their respective controls. The plant content of elements such as nitrogen required for crops was also significantly increased after the PEPV40 bacterial application; up to 15.8% under saline stress conditions.

The plant assays carried out in a chamber and the greenhouse broadly showed that *B. halotolerans* and *R. laguerreae* promoted the plant growth of escarole crops in both growth conditions. In the initial stages of growth and in vitro experiments, the escarole seedlings inoculated had longer stems than the plants of uninoculated controls, suggesting that both strains exerted a beneficial effect on plant growth and development. Moreover, in the greenhouse experiments the number of leaves was also higher, an interesting point in escarole cultivation.

Other studies with bacterial strains from the same genera have also reported significant improvements. A similar increase in leaf mass and grain yield but in fava bean plants was also detected after the application with a biofertilizer based on a *R. laguerreae* strain [31]. After the application of a *B. halotolerans* strain, El-Akhdar and collaborators [32] reported an enhancement not only in wheat plant promotion but also in nitrogen plant content. 

Finally, the results here reported indicated for the first time an improvement in the nutrient content and escarole growth after the application of a *Bacillus* and *Rhizobium* bacterial strain. Moreover, as far as we know, this was the first study performed with escarole (*Cichorium endivia* L.) crops.

### 3.4. Effects of Bacterial Strains on Plant Phenolic Composition

The analysis performed allowed for the determination of 15 different phenolic compounds in escarole leaves. To be precise, 10 phenolic acids (one hydroxybenzoic acid and nine hydroxycinnamic acid derivatives) and five flavonols (one quercetin derivative and four kaempferol derivatives) were identified and quantified in the samples (Table 3). Phenolic acids accounted around 70% of total phenolic compounds acids with cichoric acid the most abundant followed by caffeoyl-quinic acid, caffeoyl-tartaric acid and protocatechuic acid glucoside (Table 4). With regard to flavonols, this family of phenolic compounds accounted for around 30% of total phenolic compounds with kaempferol derivatives the most abundant compound and in particular the glucuronide derivative (Table 5). This phenolic composition agreed with those previous reported [5,9,17] although some compounds such as the protocatechuic acid derivative were identified in this work for the first time.

The salinity level of grown media had an important effect on the phenolic content of the escarole leaves of non-inoculated plants. In the case of both phenolic acids and flavonols (Table 4 and Table 5), the content of all of the detected compounds and therefore the total content was significantly lower when plants were grown under saline conditions than under non-saline conditions. This behavior was different from that observed in other plants such as lettuce [33] but it has been reported that the effect of salinity on the phenolic content of herbaceous plants was strongly dependent on both plant variety and the type of salinity to which it was exposed [34]. These results point out for the first time that the biosynthesis and/or accumulation of phenolic compounds in the leaves of *Cichorium endivia* L. plants was negatively affected. Thus, the different strategies that can ease those negative effects such as the use of PGPR bacteria proposed in this work need to be assessed.

The results obtained pointed out that the effect of bacterial inoculation on the phenolic composition of escarole leaves was different depending not only on the phenolic compound but also on the growth conditions and the bacterial strain employed. Under non-saline conditions, the inoculation with PEPV40 or SCCPVE07 strains led to lower levels of total phenolic acids (Table 4). Although the content of some phenolic compounds was not or only slightly affected by inoculation, when compared with the non-inoculated plants, the inoculated plants showed significantly lower levels of the most abundant phenolic compounds detected, i.e., cichoric and caffeoyl-quinic acids. On the contrary, the content of some phenolic acids such as the protocatechuic acid derivative was significantly higher in the inoculated plants, mainly when the SCCPVE07 strain was used. In fact, it was observed that the SCCPV07 bacterial genome included genes encoding enzymes involved in the protocatechuic acid pathway, which may explain the higher concentration. As for the flavonoid content (Table 5), the inoculation with SCCPV07 in plants grown under non-saline conditions led to significantly higher levels of flavonols in the escarole leaves. It seems that the inoculation with this strain favored the biosynthesis of flavonols in escarole leaves, mainly of the most abundant kaempferol derivatives (glucuronide and malonyl-glucoside derivatives). The inoculation with PEPV40 under these conditions did not significantly modify the total flavonol content of the escarole leaves although it led to significantly higher levels of kaempferol 3-*O*-malonyl glucoside, the second most important flavonol detected in the plants.

When the plants were grown under saline conditions, the inoculation with the PEPV40 strain significantly increased the total concentration of both phenolic acid and flavonols in the escarole leaves (Table 4 and Table 5). As aforementioned, the genome of this strain showed a wide variety of important genes involved in the biosynthesis pathway of flavonols and phenolics acids, which might explain those contents. In fact, the inoculation with this strain not only alleviated the negative effects that salinity had on the phenolic content but also, in the case of flavonols, led to higher contents than the control plants grown under non-saline conditions (Table 5). Thus, the use of this type of PGPR bacteria under salinity stress grown conditions could improve the escarole quality with regard to its phenolic composition.

The effect of SSCPVE07 inoculation in the plants grown under saline conditions differed depending on the type of phenolic compounds. Hence, under those conditions, the plant inoculated with this strain showed lower levels of total phenolic acids. However, slightly higher levels of flavonols (although the differences were not significant) when compared with control samples were detected in the SSCPVE07 inoculated plants. These levels were similar to those found in the control samples grown under non-saline conditions, which pointed out that the inoculation with this strain could ease the negative effects that salinity stress exerted on the accumulation of flavonols in escarole leaves.

Hence, although both PGPR bacteria strains assayed could alleviate the negative effects of salinity stress on the phenolic composition of *Cichorium endivia* L. plants, the PEPV40 strain provided the best results, even improving the phenolic contents of escarole leaves from plants grown under non-stressful conditions.

## 4. Conclusions

This study is the first report on the effects of the inoculation of escarole plants with *R. laguerreae* a *B. halotolerans* strains. The results showed how bacterial inoculation led to significant increases not only in the plant development but also in the nutritional content and food quality even under saline stress conditions. The edible parts of escarole inoculated plants showed significant increases in the content of phenolics acids such as cichoric acid and caffeoyl-tartaric acid as well as flavonoids such as kaempferol 3-*O*-glucuronide. Moreover, a relevant enhancement in nitrogen, phosphorus and magnesium content was also observed. The results showed that inoculation with *R. laguerreae* PEPV40 and *B. halotolerans* SCCPVE07 have an interesting agronomic potential, improving the content of several phenolic compounds of escarole plants. Furthermore, the bases for this type of fertilization could be established in this crop even under conditions of saline stress, maintaining food safety and increasing plant development. 

## Figures and Tables

**Table 1:** Effects of *Rhizobium laguerreae* PEPV40 and *Bacillus halotolerans* SCCPVE07 inoculation on the nutrient contents of escarole plants.

	**N (g/100 g)**				**C (g/100 g)**			
	Non-Saline Conditions		100 mM NaCl		Non-Saline Conditions		100 mM NaCl	
	Mean ± SE	%	Mean ± SE	%	Mean ± SE	%	Mean ± SE	%
Control	3.69 ± 0.10		2.90 ± 0.05		41.88 ± 0.15		35.74 ± 0.47	
PEPV40	3.58 ± 0.01	-	3.36 ± 0.01 *	+15.8	43.25 ± 0.27	+3.3	39.07 ± 0.23*	+9.3
SCCPVE07	3.24 ± 0.22	-	2.74 ± 0.05	-	41.53 ± 1.20	-	35.18 ± 0.32	-
	**P (g/100 g)**				**Mg (g/100 g)**			
	Non-Saline Conditions		100 mM NaCl		Non-Saline Conditions		100 mM NaCl	
	Mean ± SE	%	Mean ± SE	%	Mean ± SE	%	Mean ± SE	%
Control	0.60 ± 0.02		0.40 ± 0.01		0.67 ± 0.03		0.46 ± 0.01	
PEPV40	0.88 ± 0.03 *	+ 46.7	0.57 ± 0.01 *	+42.5	0.90 ± 0.07 *	+34.3	0.66 ± 0.01 *	+43.5
SCCPVE07	0.75 ± 0.01 *	+ 25.0	0.35 ± 0.02	-	1.04 ± 0.01 *	+55.2	0.38 ± 0.02	-
	**K (g/100 g)**				**Fe (mg/Kg)**			
	Non-Saline Conditions		100 mM NaCl		Non-Saline Conditions		100 mM NaCl	
	Mean ± SE	%	Mean ± SE	%	Mean ± SE	%	Mean ± SE	%
Control	2.90 ± 0.08		3.18 ± 0.07		0.51 ± 0.04		0.10 ± 0.01	
PEPV40	1.83 ± 0.09	-	2.50 ± 0.07	-	0.69 ± 0.06 *	+35.3	0.94 ± 0.03 *	+840
SCCPVE07	2.87 ± 0.04	-	3.61 ± 0.22 *	+13.5	1.14 ± 0.02 *	+123.5	0.12 ± 0.01	+20.0
	**Na (g/100 g)**							
	Non-Saline Conditions		100 mM NaCl					
	Mean ± SE	%	Mean ± SE	%				
Control	0.51 ± 0.02		4.07 ± 0.12					
PEPV40	0.30 ± 0.01 *	-	2.57 ± 0.07 *	-				
SCCPVE07	0.52 ± 0.06	+1.96	4.57 ± 0.49 *	+12.3				

* indicates a significant difference between inoculated values and the negative control, *p* ≤ 0.05 according to Fisher’s Protected LSD (least significant differences). SE = standard error.

**Table 2:** Results from in vitro growth promotion experiments and effects of *Rhizobium laguerreae* PEPV40 and *Bacillus halotolerans* SCCPVE07 inoculation on the growth and number of leaves of escarole plants under greenhouse conditions.

	**Results from In Vitro Growth Promotion Experiments**							
	Stem Length (cm)				SFW(g)			
	Non-Saline Conditions		100 mM NaCl		Non-Saline Conditions		100 mM NaCl	
	Mean ± SE	%	Mean ± SE	%	Mean ± SE	%	Mean ± SE	%
Control	14.0 ± 0.3		16.0 ± 0.2		0.33 ± 0.02		0.36 ± 0.01	
PEPV40	16.0 ± 1.0 *	+14.3	18.0 ± 3.4 *	+12.5	0.39 ± 0.03 *	+18.2	0.40 ± 0.05	+13.9
SCCPVE07	15.0 ± 0.3	+7.1	18.0 ± 0.2	+12.5	0.36 ± 0.01	+9.1	0.45 ± 0.01 *	+25.0
****	**Results from In Vitro Greenhouse Experiments**							
	SDW(g)				SFW(g)			
	Non-Saline Conditions		100 mM NaCl		Non-Saline Conditions		100 mM NaCl	
	Mean ± SE	%	Mean ± SE	%	Mean ± SE	%	Mean ± SE	%
Control	42.18 ± 0.55		27.07 ± 0.67		1.76 ± 0.02		1.13 ± 0.01	
PEPV40	49.81 ± 0.53 *	+18.1	31.18 ± 0.61 *	+15.2	2.07 ± 0.02 *	+17.6	1.30 ± 0.03 *	+15.0
SCCPVE07	43.64 ± 0.61	+3.5	47.63 ± 0.62 *	+76.0	1.82 ± 0.03	+ 3.4	1.99 ± 0.03 *	+76.1
	**Results from In Vitro Growth Promotion Experiments**				**Results from In Vitro Greenhouse Experiments**			
	Stem Length (cm)				Number of Leaves			
	Non-Saline Conditions		100 mM NaCl		Non-Saline Conditions		100 mM NaCl	
	Mean ± SE	%	Mean ± SE	%	Mean ± SE	%	Mean ± SE	%
Control	4.53 ± 0.27		4.69 ± 0.16		12.87 ± 0.44		12.60 ± 0.43	
PEPV40	5.43 ± 0.15	+19.9	4.71 ± 0.11	+0.4	14.67 ± 0.47 *	+14.0	12.47 ± 0.39	-
SCCPVE07	4.61 ± 0.21	+1.8	5.34 ± 0.11 *	+13.9	14.47 ± 0.61 *	+12.4	13.20 ± 0.39	+4.8

* indicates a significant difference between inoculated values and the negative control, *p* ≤ 0.05 according to Fisher’s Protected LSD (least significant differences). SE = standard error. SFW = shoot fresh weight. SDW = shoot dry weight.

**Table 3:** Chromatographic, spectral and spectrometric features of the phenolic compounds identified in escarole plants.

Compound	t_R_ (min)	UV ʎ_max_ (nm)	[M–H] (*m*/*z*)	Fragment Ions (*m*/*z*)	Identification
1	10.58	284	315	153	Protocatechuic acid glucoside
2	11.09	332–295 (sh)	311	179,135	Caffeoyl-tartaric acid
3	12.24	338–292 (sh)	343	179,135	Caffeic acid derivative
4	14.01	328–300 (sh)	353	191	Caffeoyl-quinic acid
5	15.24	328–300 (sh)	353	191	Caffeoyl-quinic acid
6	16.8	324–296 (sh)	179	135	Caffeic acid
7	17.3	328–300 (sh)	295	179,135	Caffeoyl-malic acid
8	21.37	328–300 (sh)	473	311,293,179,149,135	Cichoric acid
9	27.9	-	477	301	Quercetin 3-*O*-glucuronide
10	29.34	328–300 (sh)	487	307,293,193,179,135	Caffeoyl-ferouyl-tartaric acid
11	29.9	-	572	461,397,285	Kaempferol glucuronide derivative
12	30.78	328–300 (sh)	515	353,191,179,135	Dicaffeoyl-quinic acid
13	32.6	348–296 (sh)	461	285	Kaempferol 3-*O*-glucuronide
14	33.06	348–296 (sh)	447	285	Kaempferol 3-*O*-glucoside
15	36.00	348–296 (sh)	533	489, 285	Kaempferol 3-*O*-malonyl-glucoside

**Table 4:** Concentration, expressed as mean ± standard deviation, of phenolic acids of escarole plants.

	Non-Saline Conditions (g Kg^−1^)			100 mM NaCl (g Kg^−1^)		
	Control	PEPV40	SCCPVE07	Control	PEPV40	SCCPVE07
Protocatechuic acid glucoside	8.39 ± 0.06 c	13.28 ± 0.06 b	14.0 ± 0.1 a	3.8 ± 0.2 b, *	6.42 ± 0.07 a, *	3.11 ± 0.06 c, *
Caffeoyl-tartaric acid	6.10 ± 0.03 a	4.51 ± 0.01 c	5.85 ± 0.06 b	4.9 ± 0.3 b, *	6.45 ± 0.08 a, *	3.94 ± 0.02 c, *
Caffeic acid derivative	0.43 ± 0.02 a	0.43 ± 0.02 a	0.43 ± 0.03 a	0.41 ± 0.02 b	0.53 ± 0.01 a, *	0.381 ± 0.003 b
Caffeoyl-quinic acids ^1^	13.21 ± 0.05 a	10.40 ± 0.07 b	8.58 ± 0.02 c	8.9 ± 0.5 b, *	12.97 ± 0.08 a, *	9.325 ± 0.008 b, *
Caffeic acid	0.64 ± 0.01 a	0.53 ± 0.03 c	0.582 ± 0.003 b	0.48 ± 0.02 b, *	0.67 ± 0.006 a, *	0.436 ± 0.009 c, *
Caffeoyl-malic acid	0.60 ± 0.01 a	0.46 ± 0.02 b	0.45 ± 0.03 b	0.41 ± 0.01 b, *	0.59 ± 0.01 a, *	0.37 ± 0.01 c, *
Cichoric acid	37.1 ± 0.2 a	24.8 ± 0.2 c	25.76 ± 0.09 b	24.56 ± 1.35 b, *	34.51 ± 0.04 a, *	23.38 ± 0.07 b, *
Caffeoyl-ferouyl-tartaric acid	1.21 ± 0.03 a	0.47 ± 0.02 c	1.03 ± 0.04 b	1.51 ± 0.07 b, *	2.50 ± 0.07 a, *	0.82 ± 0.03 c, *
Dicaffeoyl-quinic acid	1.37 ± 0.06 a	0.81 ± 0.03 b	1.42 ± 0.02 a	1.35 ± 0.06 a	1.23 ± 0.04 b, *	0.94 ± 0.02 c, *
Total phenolic acids	69.0 ± 0.1 a	55.7 ± 0.5 c	58.1 ± 0.3 b	46 ± 2 b, *	65.87 ± 0.07 a, *	42.71 ± 0.08 c, *

^1^ Sum of caffeoyl quinic acids. Different letters within each type of conditions (saline or non-saline) and each row indicates Scheme 0. * indicates significant differences (*p* ≤ 0.05 according to Fisher’s Protected LSD) between the saline sample and the corresponding non-saline sample.

**Table 5:** Flavonol composition of escarole plants, expressed as mean ± standard deviation.

	Non-Saline Conditions (mg/g)			100 mM NaCl (mg/g)		
	Control	PEPV40	SCCPVE07	Control	PEPV40	SCCPVE07
Kaempferol 3-*O*-glucoside	2.20 ± 0.08 b	2.18 ± 0.01 b	2.64 ± 0.05 a	2.12 ± 0.11 b	2.64 ± 0.05 a, *	2.16 ± 0.07 b, *
Kaempferol 3-*O*-glucuronide	15.58 ± 0.10 b	15.30 ± 0.09 b	18.86 ± 0.06 a	13.60 ± 0.45 b	19.76 ± 0.06 a	14.12 ± 0.04 b, *
Kaempferol-glucuronide derivative	1.19 ± 0.03 a	0.77 ± 0.03 b	0.80 ± 0.02 b	0.97 ± 0.07 b, *	1.45 ± 0.06 a, *	1.00 ± 0.04 b, *
Kaempferol 3-*O*-malonyl-glucoside	4.95 ± 0.08 c	5.42 ± 0.08 b	5.91 ± 0.09 a	4.1 ± 0.3 b, *	5.11 ± 0.06 a, *	4.90 ±0.09 a, *
Quercetin 3-*O*-glucuronide	0.31 ± 0.01 a	0.23 ± 0.02 b	0.29 ± 0.01 a	0.21 ± 0.02 b, *	0.303 ± 0.009 a, *	0.23 ± 0.01 b, *
Total flavonoids	24.2 ± 0.4 b	23.9 ± 0.3 b	28.5 ± 0.1 a	21 ± 1 b, *	29.3 ± 0.1 a, *	22.4 ± 0.3 b, *

Different letters within each type of conditions (saline or non-saline) and each row indicates significant differences (*p* ≤ 0.05). * indicates significant differences (*p* ≤ 0.05 according to Fisher’s Protected LSD) between the saline sample and the corresponding non-saline sample.

## Data Availability

Not applicable.
